# Primary care staff's views and experiences related to routinely advising patients about physical activity. A questionnaire survey

**DOI:** 10.1186/1471-2458-6-138

**Published:** 2006-05-23

**Authors:** Flora Douglas, Nicola Torrance, Edwin van Teijlingen, Serena Meloni, Ann Kerr

**Affiliations:** 1 Department of Public Health, University of Aberdeen, Polwarth Buildings, Forresterhill, Aberdeen, AB25 2ZD, Scotland, UK; 2 NHS Health Scotland, Woodburn House, Canaan Lane, Morningside, Edinburgh, EH10 4SG, Scotland, UK; 3 Department of General Practice, University of Aberdeen, Forresterhill Health Centre, Aberdeen, AB25 2AY, Scotland, UK

## Abstract

**Background:**

United Kingdom public health policy has recently re-emphasised the role of primary health care professionals in tackling increasing levels of physical inactivity within the general population. However, little is known about the impact that this has had in practice. This study explores Scottish primary care staff's knowledge, attitudes and experiences associated with advising patients about physical activity during routine consultations.

**Methods:**

A cross-sectional questionnaire survey of general practitioners (or family physicians), practice nurses and health visitors based in four health regions was conducted during 2004. The main outcome measures included: (i) health professionals' knowledge of the current physical activity recommendations; (ii) practice related to routine physical activity advising; and (iii) associated attitudes.

**Results:**

Questionnaires were returned by 757 primary care staff (response rate 54%). Confidence and enthusiasm for giving advice was generally high, but knowledge of current physical activity recommendations was low. In general, respondents indicated that they routinely discuss and advise patients about physical activity regardless of the presenting condition. Health visitors and practice nurses were more likely than general practitioners to offer routine advice.

Lack of time and resources were more likely to be reported as barriers to routine advising by general practitioners than other professional groups. However, health visitors and practice nurses were also more likely than general practitioners to believe that patients would follow their physical activity advice giving.

**Conclusion:**

If primary health care staff are to be fully motivated and effective in encouraging and supporting the general population to become more physically active, policymakers and health professionals need to engage in efforts to: (1) improve knowledge of current physical activity recommendations and population trends amongst frontline primary care staff; and (2) consider the development of tools to support individual assessment and advice giving to suit individual circumstances. Despite the fact that this study found that system barriers to routine advising were less of a problem than other previous research has indicated, this issue still remains a challenge.

## Background

The benefits of regular physical activity (PA) include improved physical and mental health, as well as an enhanced quality of life [1-3]. Yet, despite the generally high levels of awareness of the benefits of being active within the general population, there is continuing evidence of declining levels of PA both in England and Scotland [4]. This trend has been strongly linked to increasing levels of coronary heart disease, type II diabetes, osteoporosis, colon cancer and obesity. Currently, national recommendations stipulate that adults should accumulate a minimum of 30 minutes of moderate level PA (the equivalent of brisk walking) most days of the week (30 mins PA ×5) [5].

Whilst it is recognised that policies recommending that general practitioners (GPs) promote physical activity are not universally accepted [6,7], many consider that primary care (PC) health professionals are well placed to encourage both increased levels of PA within the general population, as well as with high-risk patients [8,9]. In addition, it has been reported that PA can alleviate the symptoms of mental health illness, and that it has been found that GPs have been generally reluctant to counsel those patients suffering from mental health problems to consider increasing their PA levels to ameliorate their condition [3].

Health professionals working in PC settings are considered an important part of a wider public health drive to encourage more people to become active [5]. A recently published systematic review of the effectiveness of public health interventions aimed at increasing PA among apparently healthy adults indicated that there is some evidence of short-term changes in PA levels than can be attributed to brief advice given by a health professional [10]. This work also found that longer-term change may result if the individual is referred to an exercise specialist in the community. In addition, there is some evidence to suggest that PC-based exercise referral schemes may result in short-term impacts on PA levels for certain population groups, in particular the 'almost-active' [11].

In championing the role of PC in motivating and supporting the general population to become more physically active, Taylor highlighted the need to understand the range of multilevel and multi-sectoral factors (based on a whole systems approach) that influence the effectiveness of any health promotion programme or intervention [9]. Based on the PROCEED PRECEED health promotion planning model [12] he argued that an investigation of the PC consultation needed to include consideration of three key dimensions including: (1) predisposing factors; which relate to attributes and characteristics of the patients; (2) reinforcing factors which the PHC staff member brings to the consultation; and (3) enabling factors which relate the availability of resources, referrals, rules and protocols, and service structures.

Reinforcing factors in this context relate to the attitudes, knowledge, beliefs and behaviours of those professionals regarded as instrumental in motivating and supporting patients to become more physically active, and Taylor argued that there has been a lack of research in this area [9]. Steptoe *et al*. [13] also maintained that it is crucial to understand the beliefs and practice of those primary health professionals (working at the frontline) to fully appreciate and understand if public health policy changes have been implemented effectively.

To date, there is a little Scottish empirical data available to policy makers about such reinforcing factors [14]. The few studies that have been conducted elsewhere found that GPs were more comfortable discussing PA with high-risk groups, especially when linked to exercise prescription schemes [15]. Factors preventing routine PA advising included: lack of time; lack of financial reimbursement; relevance to patient's presenting condition; perceptions of poor patient compliance; lack of confidence in providing advice; insufficient knowledge about the benefits of exercise; and a lack of appropriate tools to assess and prescribe exercise [16-20]. Lack of self-efficacy associated with ability to motivate patients and achieve the desired outcome has also been identified [21,22] as another barrier. Typically, studies have failed to explore the views and practice of the range of health professionals groups within the same study.

### Aims and objectives

Our study investigated attitudes, current practice and knowledge of Scottish PC staff related to advising patients about PA during routine consultations. Staff groups included in this survey included GPs, health visitors (HV) and practice nurses (PN).

## Methods

A questionnaire was developed following a review of the literature, with selected questions taken from a previous study [18]. Four health board regions in Scotland, (from a possible 15) were selected for the study to reflect a cross section of urban, remote and rural regions. Following a pilot study, the questionnaire was mailed to all GPs (802) based in all 180 practices in the four health board areas in Scotland, as well as 317 PNs and 289 HVs. A mailing list for all principal GPs in each of the four health regions was obtained from ISD (Information Services Division) Scotland. The questionnaire was personally addressed to each GP and then generically to the PN and HV in each practice (no personalised mailing list was available for these professional groups). Reminders were posted after three weeks. Data was analysed using SPSS ver12.0. Chi-squared tests were used to test for associations between categorical data. Normally distributed continuous data were analysed using were using t-tests. Ethical approval was granted by the Scotland (A) Multiple Research Ethics Committee.

## Results

### Response rates

Questionnaires were returned by 757 (54%) of PC staff. This included 376 (47%) GPs, 212 (67%) PNs and 169 (59%) HVs. At least one GP in 80% of the PC practices returned a completed questionnaire. The GP responders were very similar to the national picture in terms of age, sex and qualifications [18].

Information provided by ISD Scotland about all GPs included in the study indicated that there were no significant differences between GP responders and non-responders for age (t-test, p = 0.78) or gender (chi-square test, p = 0.38). There were also no significant differences in the response rates from single-handed practices compared to partnerships (chi-square, p = 0.75).

### Knowledge about current recommendations for sedentary adults

Only 13% of GPs (n = 49), 9% of HVs (n = 15) and 7% of PNs (n = 15) correctly described the current recommendations (i.e. accumulation of 30 mins PA ×5 weekly, including frequency, duration and intensity), while 18% of GPs (n = 68), 12% of HVs (n = 21) and 10% of PNs (n = 22) recorded the previous recommendations (i.e. 20 mins × 3/week). However, approximately a third from each group correctly identified at least one component of the current recommendations.

### Perceptions of PA levels within the general population

**Figure 1:**
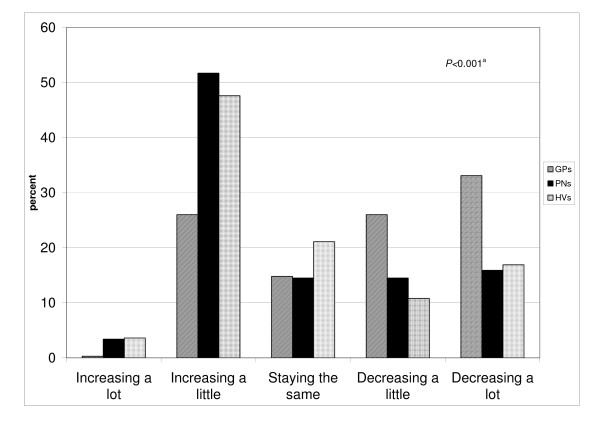
**Primary care staffs' perceptions of overall physical activity levels (%)**. Note: GP denotes General Practitioner, PN denotes Practice Nurse and HV denotes Health Visitor ^a ^from Pearson χ^2 ^test.

We found a significant difference in the opinions of PC staff about levels of PA amongst the general population within Scotland. More PNs and HVs than GPs thought overall PA levels were increasing (Figure 1).

### Advice given during consultations with adult patients who are apparently healthy

**Figure 2:**
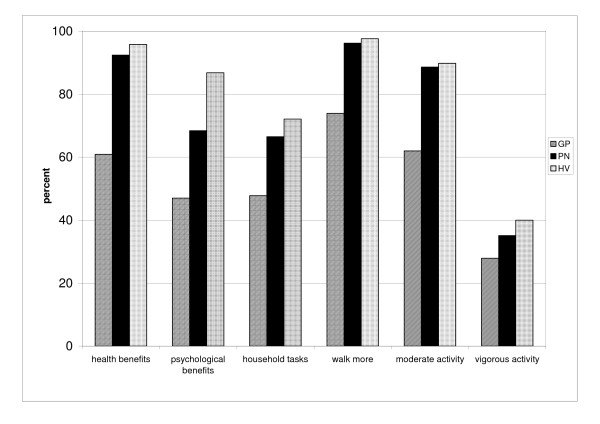
**Topics likely to be covered during consultations with apparently healthy adult (%)**. Note: GP denotes General Practitioner, PN denotes Practice Nurse and HV denotes Health Visitor.

There were significant differences in respondents' advising practice both in terms of whether they discussed PA in the first place, and about the types of advice they gave. Overall, PNs and HVs were more likely to say they gave all types of PA advice compared to GPs (Figure 2). For example, 62% GPs indicated they were very likely or likely to recommend all apparently health adult patients take moderate exercise compared to 88% HVs and 90% PNs. However, the majority in all professional groups were all unlikely to recommend vigorous activity. An overwhelming majority recommended walking (85% – 98%) as the most popular form of exercise.

### Advice given for specific medical conditions

Overall GPs and PNs gave advice more often than HVs for most medical conditions. PNs consistently "always" give advice for every condition except for depression. Conversely, four out of five HVs "always or often" give advice for depression (almost as often as they gave advice to overweight patients). All PC staff were more likely to "always" provide PA advice for overweight patients than for any other condition (Table 1).

### Attitudes associated with health promotion and PA advising

Overall, most respondents agreed that health promotion was an important part of their work, and that promoting PA was a key part of PC. In addition, the majority of all PC staff thought they had sufficient knowledge to advise on the issue.

GPs were more likely to agree that they advised patients about PA only if it was linked to the presenting condition, while PNs and HVs were more likely to encourage most patients to increase their PA levels. Paradoxically, very few respondents in each group agreed that they only discussed PA if the patient raised the issue (Table 2).

### Perceived barriers to giving routine PA advice to patients

When asked to think about factors that prevent discussion of PA, GPs regarded lack of time as more of a barrier than PNs or HVs did, and more GPs (23%) than PNs (3%) or HVs (5%) indicated that a financial incentive might change practice. However, 40 to 60% of all respondents agreed that educational materials are insufficient for their needs, and approximately half thought there was a lack of specific training available for health professionals, despite the fact that they indicated they had sufficient knowledge to advise on PA (Table 3). Curiously, more GPs than PNs and HVs thought that patients were unlikely to be motivated to follow their advice (30.7% vs 13.8% vs 12.0% respectively).

## Discussion

### Reinforcing factors

#### Knowledge

The relatively low levels of knowledge and accuracy in describing the current PA guidelines were surprising, particularly given that the recommendations for sedentary adults are currently present in several national guidelines, such as, management of lipids and prevention of coronary heart disease, and diabetes management [23,24]. Furthermore, surprisingly high numbers of HVs and PNs thought that levels of PA were increasing in the population. Most GPs believed the reverse was true. One can question whether it is possible that this finding has arisen due to the context within which the patient consultation occurs, i.e. where patients maybe more likely to disclose their PA experiences in a less formal and time pressured atmosphere. If this was the case, one might argue that GPs would be less likely to hear patients' accounts of their attempts at PA behaviour, compared to PNs and HVs. However, this apparent lack of knowledge about PA recommendations and current population trends is concerning, as, well-informed health professionals are considered essential for effective delivery of PC-based health promotion [25].

#### Advising practice

Most PC staff said they currently advised patients about PA during routine consultations. However, there was variation in practice between HVs and PNs on the one hand and GPs on the other. For example, HVs and PNs (~90%) were more likely to intervene with advice than GPs (~70%). It is possible that this effect could be explained by the finding that also indicated that GPs were more likely to discuss PA if they perceived it as relevant to a patient's presenting condition.

All PC groups were more likely to intervene with PA advice if they thought the patient was overweight. Proportions of respondents who advised staff for other conditions was generally very low. These findings are consistent with other studies [16,17]. However, HVs were more likely to discuss PA with patients suffering from depression and discuss psychological benefits than GPs or PNs. This difference is interesting in light of the findings reported by the Mental Health Foundation, which also indicated that very few GPs considered providing advice about exercise for those with mild to moderate depression [3].

Walking was the most popular form of exercise recommended by all groups. This is encouraging, as walking advice is considered most relevant for those who are currently sedentary, and/or are from lower socio-economic backgrounds; who also consistently report lower levels of PA [26].

While it is reassuring to observe that PC staff appear to be giving generally sound advice, for example, most were unlikely to advise patients to take vigorous PA – there could be more clarity about the message given, particularly as the correct advice currently exists in clinical guidelines. However, the lack of use or adherence to clinical guidelines amongst GPs remains a challenge [27].

#### Attitudes

The majority of study respondents expressed positive views about health promotion as a core aspect of PC; with 90% of all respondents indicating that they believed PA promotion in PC was important. In addition, the majority indicated they would discuss PA with their patients, even if it was not raised during the consultation. These findings indicate that there is good practitioner support for PA promotion in PC.

Whilst respondents had an apparent lack of knowledge about the current guidelines, paradoxically, most thought they had sufficient knowledge to promote PA with their patients. Perhaps these apparently contradictory findings indicate a general lack of awareness or understanding about current behaviour change approaches to counselling. Nevertheless, it is important to raise awareness amongst each professional group about current PA recommendations, preferably in the context of increased education and training opportunities, which study respondents also indicated they needed.

While GPs were more inclined to mention lack of remuneration as a barrier than PNs or HVs, less than one in four agreed with this statement. The fact that there are significant differences between GPs and nurses about this issue is perhaps not surprising given the different methods of remuneration for each group. Moreover, our findings suggest that GPs were less likely to be motivated by financial rewards than other studies have indicated [17].

In terms of other significant differences in study groups' views, lack of time was considered more of a barrier to routine PA advising for GPs than it was for PNs and HVs. However, only 50% of GPs thought lack of time was a barrier compared to 93% in a similar study [18]. It is possible that this is a secular effect brought about by the changes in PC priorities over the last five years, which have witnessed an increased emphasis on health promotion and health improvement.

Another perceived barrier worth highlighting is that GPs were less likely to think that patients would be motivated to follow their advice compared to HVs and PNs. This may be indicative of higher levels of self-efficacy related to patient motivation in the nurse groups compared to GPs. Low GP self-efficacy has also featured in other studies [20-28]. However, further study is needed to investigate why this is the case, and if those who perceive themselves to be likely to motivate patients to change their behaviour are effective in doing so.

### Predisposing and enabling factors

This study focussed on individual level or 'reinforcing factors' [12] associated with PC professionals' attitudes and beliefs associated with PA promotion. However, if PC is to be effective in embedding policy within practice, other dimensions such as patient characteristics, so-called 'predisposing factors' and, the system capability or 'enabling factors' such as service structures, resources, protocols [29] need to be considered.

It is essential to understand patients' perspectives, particularly about the relevance, acceptability and impact of PC staff intervening with PA advice during routine consultation. Some qualitative studies are starting to shed a little light on these issues. We know for instance that patients regard GPs as a credible source of advice, and some do seek their GP's help with gaining access to exercise referral schemes [30]. However, evidence suggests that these individuals may be actively contemplating becoming more physically active in the first place [11]. It may be possible that other patient groups believe GPs have the necessary knowledge, but limited time in which to routinely advise them on the issue, and are therefore not raising it during a consultation with their doctor. Nevertheless, evidence about patient's perspectives is scarce. Further patient-centred research may also yield data about the validity (or otherwise) of each of the respective professional group's views about their efficacy in motivating patients to be more physically active.

Our findings concur with Eakin and colleagues [29] who found in their review of physician barriers to PA counselling provision, that practitioner reports of system barriers (e.g. lack of time, resources, training and protocols or guidelines) continue to feature alongside individual level barriers associated with PA promotion, such as not having the requisite knowledge, skills, efficacy etc, needed to motivate patients to change. They maintain that the development of PC based, PA interventions must therefore take account of organisational systems and structures if such initiatives can feasibly be adopted and implemented by busy staff working in the real world. They and others [31,32] also highlight the need to develop tools and guidelines to support staff to promote PA. Our that respondents believe that there is a lack of educational tools and training for staff to support individual practice, but it was beyond the scope of the study to investigate such issues in detail and further work is required to identify what is required.

### Strengths and limitations of the study

The survey achieved a reasonable response rate given the timing of the survey, which coincided with the new GP contract in the UK. Another study of GPs' beliefs and behaviour related to health promotion gained a 48% response rate [13]. However, we also believe that this is the largest survey of its kind of PC professionals from any one health care system.

It is possible that the generally very positive responses to questions associated with the relevance of this issue within PC reflect the views of the "enthusiasts". However, the demographic characteristics of our GP respondents are similar to the national picture, and the characteristics of the GP non-responders were similar to the responders, suggesting that our sample is representative.

Our study was also limited to these particular study groups for pragmatic reasons. Clearly there are other health professionals working in PC whose beliefs and practice related to this issue should also be understood. Nevertheless this is the first study to our knowledge that has attempted to capture a range of PC staff in the same survey on this issue.

Whilst there are clearly some similarities between ours and others findings e.g. barriers to routine advising related to a perceived lack of time and relevance to the patient's presenting condition, there were differences related to the perceived role financial remuneration could play in encouraging routine PA advising amongst GPs. In addition, other studies did not ask respondents about their current knowledge and beliefs about current PA recommendations, or their perceptions about the general levels of PA in the population. We believe these factors may have a bearing on whether staff will intervene with advice (or not) and the nature of the advice that is given.

## Conclusion

The current state of respondents' knowledge related to the PA recommendations is of concern, particularly when most respondents thought they had sufficient knowledge to promote this issue. However, study respondents also reported a lack of appropriate training and development opportunities for health professionals.

At the same time, the majority was supportive of PA promotion as a core aspect of PC, indicating a positive foundation in which to develop further work in this area. However, there does not appear to be a pattern of systematic assessment and practice related to giving PA advice across each study group. It is important that patients receive clear and consistent messages about PA from the wide range of health professionals working within PC as a routine aspect of healthcare. The development of tools and guidelines may assist staff to provide consistent, accurate and appropriate advice tailored to individual patient needs. Training and development activities may also help to raise awareness about related issues. Financial remuneration alone may not be a sufficient incentive to encourage more GPs to promote PA within their practice.

It was surprising to find that HVs and PNs were more likely than GPs to report that they gave PA advice routinely, while at the same time believing population levels of PA to be increasing. It is possible that small groups of patients are increasingly discussing the issue with their HV or PN, where time constraints appear to be less of an issue than for GPs, making it easier for patients to discuss their PA behaviour. However, it was beyond the scope of this study to explore these findings in detail, and it certainly requires further investigation. It is particularly important to understand this issue more clearly when one also considers the lower levels of knowledge about current PA recommendations, amongst the nursing groups, but their generally higher levels of support and enthusiasm for PA promotion compared to GPs. With the expanding public health role of nurses within PC, it seems there is even more potential to reach a large proportion of the UK population. However as we stressed earlier, it is crucial that the advice provided throughout is accurate, credible and consistent.

Nursing groups in also appeared to believe they were more likely to motivate patients to comply with their PA advice than GPs. Why is GP self-efficacy about this issue low and what can be done to enhance it? These are important questions for policy makers, which need to be considered seriously if the health improvement policy aspiration about making PA promotion a routine aspect of primary health care can be realised. For, as evidence on the dynamics of motivation suggests, those who do not expect positive outcomes from engaging in certain behaviours have no motivation to invest in them [33].

This study was limited to an exploration of the beliefs, attitudes and practice of some of the health professional groups who work in PC. It would be useful to have data about other relevant groups not covered by this study, e.g. community-based physiotherapists and district nurses, given their combined potential to reach an even larger section of the population with PA advice. Furthermore, this work is based on self-reported behaviour. Future studies might benefit from the inclusion of observational methods to validate claims of practice. In addition, in order to enhance the efficacy of PC-based approaches to PA promotion there is also a need to investigate the role that predisposing patient attitudes and beliefs, and, existing system or organisational capacity and mechanisms (e.g. protocols and guidelines) plays.

Finally, policymakers, respective professional bodies and health improvement agencies should consider addressing these findings, nationally and locally, if public health policy aims about fully engaging PC staff in efforts to encourage and support the general population to be more physically active are to be effective.

## Competing interests

The author(s) declare that they have no competing interests.

## Authors' contributions

FD was responsible for designing the study, interpreting the findings and writing the paper.

NT was responsible for data collection, analysis and interpretation and writing the paper.

EvT was responsible for designing the study, interpreting the findings and writing the paper.

SM was responsible for commissioning the study, and contributed to the interpretation of the findings and writing the paper.

AK was responsible for commissioning the study, contributed to interpreting the findings and commenting on drafts of this paper.

## Pre-publication history

The pre-publication history for this paper can be accessed here:



## Figures and Tables

**Table 1:** Frequency in providing physical activity advice for specific medical conditions, n (%)

		**Always**	**Often**	**Occasionally**	**Never**	**p-value^1^**
Overweight						
	**GP**	224 (60)	137 (37)	10 (3)	0	<0.001
	**PN**	165 (79)	43 (21)	2 (1)	0	
	**HV**	90 (54)	54 (33)	22 (13)	0	

Hypertension						
	**GP**	154 (42)	173 (47)	43 (12)	1 (0.3)	<0.001
	**PN**	144 (68)	60 (28)	7 (3)	0	
	**HV**	51 (32)	51 (32)	43 (27)	15 (9)	

Known ischaemic heart disease						
	**GP**	137 (37)	191 (52)	43 (12)	0	<0.001
	**PN**	112 (53)	74 (35)	20 (10)	4 (2)	
	**HV**	58 (35)	51 (31)	34 (21)	21 (13)	

Diabetes						
	**GP**	131 (35)	166 (45)	68 (18)	6 (2)	<0.001
	**PN**	140 (66)	51 (24)	19 (9)	1 (1)	
	**HV**	31 (20)	39 (25)	60 (39)	25 (16)	

Hypercholesteroleamia						
	**GP**	96 (26)	157 (42)	108 (29)	9 (2)	<0.001
	**PN**	113 (54)	73 (35)	23 (11)	1 (1)	
	**HV**	46 (30)	40 (26)	45 (29)	25 (16)	

Depression						
	**GP**	74 (20)	167 (45)	114 (31)	15 (4)	<0.001
	**PN**	39 (19)	85 (41)	70 (34)	14 (7)	
	**HV**	74 (44)	61 (36)	28 (17)	5 (3)	

Note: GP denotes General Practitioner, PN denotes Practice Nurse and HV denotes Health Visitor

^1 ^from Pearson χ^2 ^test

**Table 2:** Primary care staff's opinions on promoting physical activity, n (%)

		**Strongly agree**	**Agree**	**Neither agree nor disagree**	**Disagree/Strongly disagree**	***p*-value^1^**
Health promotion is an important part of primary care work	**GP**	158 (42)	191 (51)	24 (6)	3 (1)	<0.001
	**PN**	163 (77)	46 (22)	2 (1)	0	
	**HV**	155 (92)	14 (8)	0	0	

Promoting physical activity is important in primary care	**GP**	135 (36)	211 (56)	26 (7)	4 (1)	<0.001
	**PN**	138 (66)	68 (33)	2 (1)	1 (1)	
	**HV**	131 (78)	37 (22)	0	0	

I only advise patients about physical activity if linked to their presenting problem	**GP**	16 (4)	163 (43)	75 (20)	122 (33)	<0.001
	**PN**	11 (5)	56 (27)	31 (15)	113 (53)	
	**HV**	6 (4)	34 (20)	21 (13)	107 (64)	

I have sufficient knowledge to advise patients about physical activity	**GP**	44 (12)	204 (54)	94 (25)	34 (9)	0.02
	**PN**	19 (9)	130 (62)	42 (20)	19 (9)	
	**HV**	29 (18)	103 (62)	25 (15)	9 (5)	

I try to encourage as many patients as possible to increase their physical activity	**GP**	67 (18)	190 (51)	83 (22)	36 (10)	<0.001
	**PN**	71 (34)	112 (53)	24 (11)	4 (2)	
	**HV**	60 (36)	92 (55)	10 (6)	5 (3)	

I only discuss physical activity if the patient mentions it	**GP**	1 (0.3)	20 (5)	46 (12)	308 (82)	0.17
	**PN**	2 (1)	5 (2)	18 (9)	185 (88)	
	**HV**	0	4 (2)	18 (11)	146 (87)	

Note: GP denotes General Practitioner, PN denotes Practice Nurse and HV denotes Health Visitor

^1 ^from Pearson χ^2 ^test

**Table 3:** Reasons given by primary care staff about why they do not give advice to patients about physical activity, n (%)

		**Strongly agree/agree**	**Neither agree nor disagree**	**Disagree/strongly disagree**	***p*-value **^1^
Patients are unlikely to be motivated to follow advice to be more active	**GP**	115 (31)	78 (21)	81 (49)	<0.001
	**PN**	29 (14)	46 (22)	135 (64)	
	**HV**	20 (12)	30 (18)	116 (70)	
I don't have enough time to promote physical activity to my patients	**GP**	181 (48)	65 (17)	128 (34)	<0.001
	**PN**	44 (21)	27 (13)	139 (66)	
	**HV**	16 (10)	20 (12)	129 (78)	
I would be more likely to promote physical activity if there was a financial incentive	**GP**	85 (23)	80 (21)	208 (56)	<0.001
	**PN**	7 (3)	26 (12)	177 (84)	
	**HV**	7 (4)	10 (6)	149 (90)	
Educational materials for patients are insufficient	**GP**	213 (57)	86 (23)	76 (20)	<0.001
	**PN**	83 (39)	57 (27)	71 (33)	
	**HV**	72 (43)	29 (18)	65 (39)	
Patients expect drug treatments when they visit their GP practice	**GP**	141 (37)	85 (23)	149 (40)	0.067
	**PN**	67 (32)	64 (31)	79 (38)	
	**HV**	73 (44)	41 (25)	53 (32)	
There is a lack of available education for health professionals regarding physical activity promotion	**GP**	175 (47)	96 (26)	103 (28)	0.361
	**PN**	93 (46)	56 (27)	60 (29)	
	**HV**	87 (52)	31 (19)	48 (29)	

Note: GP denotes General Practitioner, PN denotes Practice Nurse and HV denotes Health Visitor

^1 ^from Pearson χ^2 ^test
